# Satellite Cells CD44 Positive Drive Muscle Regeneration in Osteoarthritis Patients

**DOI:** 10.1155/2015/469459

**Published:** 2015-06-01

**Authors:** Manuel Scimeca, Elena Bonanno, Eleonora Piccirilli, Jacopo Baldi, Alessandro Mauriello, Augusto Orlandi, Virginia Tancredi, Elena Gasbarra, Umberto Tarantino

**Affiliations:** ^1^Anatomic Pathology Section, Department of Biomedicine and Prevention, University of Rome “Tor Vergata”, Via Montpellier 1, 00133 Rome, Italy; ^2^“Multidisciplinary Study of the Effects of Microgravity on Bone Cells” Project, Italian Space Agency (ASI), Spatial Biomedicine Center, Via del Politecnico snc, 00133 Rome, Italy; ^3^Department of Orthopedics and Traumatology, “Tor Vergata” University of Rome, “Policlinico Tor Vergata” Foundation, Viale Oxford 1, 00133 Rome, Italy; ^4^School of Specialization in Orthopaedics and Traumatology, “Tor Vergata” University of Rome, “Policlinico Tor Vergata” Foundation, Viale Oxford 1, 00133 Rome, Italy; ^5^Department of Systems Medicine, University of Roma Tor Vergata, Via Montpellier 1, 00133 Rome, Italy

## Abstract

Age-related bone diseases, such as osteoarthritis and osteoporosis, are strongly associated with sarcopenia and muscle fiber atrophy. In this study, we analyzed muscle biopsies in order to demonstrate that, in osteoarthritis patients, both osteophytes formation and regenerative properties of muscle stem cells are related to the same factors. In particular, thanks to immunohistochemistry, transmission electron microscopy, and immunogold labeling we investigated the role of BMP-2 in muscle stem cells activity. In patients with osteoarthritis both immunohistochemistry and transmission electron microscopy allowed us to note a higher number of CD44 positive satellite muscle cells forming syncytium. Moreover, the perinuclear and cytoplasmic expression of BMP-2 assessed by *in situ* molecular characterization of satellite cells syncytia suggest a very strict correlation between BMP-2 expression and muscle regeneration capability. Summing up, the higher BMP-2 expression in osteoarthritic patients could explain the increased bone mineral density as well as decreased muscle atrophy in osteoarthrosic patients. In conclusion, our results suggest that the control of physiological BMP-2 balance between bone and muscle tissues may be considered as a potential pharmacological target in bone-muscle related pathology.

## 1. Introduction

Age-related bone diseases, as osteoarthritis and osteoporosis, are strongly associated with sarcopenia and specific muscle fiber atrophy [1, 2].

Osteoarthritis is the most common form of arthritis with increasing incidence due to elderly characterized by whole-joint involvement that leads to cartilage loss and, eventually, joint failure [3]. Damaged cartilage is replaced by a mixture of immature and disarranged collagen fibers. The subchondral bone loses its coating and is directly subjected to abnormal stress, which can lead to microfractures and formation of reparative sclerotic bone with a widely disorganized trabecular pattern [4].

Skeletal muscle is capable of interfering with bone activity, although cellular and molecular muscle-bone cross talk are not well understood yet [5]. Sarcopenia is due to an important imbalance between synthesis and deterioration of muscle proteins and cells, with a resulting poor muscular quality. This depletion strongly influences bone tropism and affects mobility and skeletal features during osteoporosis and osteoarthritis. We know that IGF-1, myostatin, TGF-b, and BMPs are factors released from muscle that have a role in bone metabolism and* vice versa*. Potential mechanisms involved in the reduction of skeletal muscle mass during sarcopenia converge on the failure of satellite cells in replacing and repairing damaged muscle fibers [6, 7]. Whether satellite cells number decreases or not, their function is generally reduced in aging. However, an important cause for reduced satellite cell function may be a result of altered systemic factors that influence and/or regulate satellite cell activity and differentiation [8].

CD44 is a transmembrane protein that plays a role in cell-cell interactions and motility in a number of cell types such as for myoblast differentiation and fusion. Indeed, CD44 plays a functional role in early myogenesis. Mylona et al. showed a transient delay of the repair after local injury in mouse tibialis anterior muscles in CD44 (−/−) mice. In a differentiation-inducing in vitro environment, a delayed myotube formation by primary myoblasts of CD44 (−/−) mice suggests an intrinsic role for CD44 [9].

Bone morphogenetic proteins (BMPs) are members of the transforming growth factor beta superfamily of cytokines. These molecules are secreted growth factors and are commonly categorized according to their structural properties; they have been extensively studied focusing on their capability to induce bone precursor cells differentiation [10].

In this retrospective study we analyzed muscle biopsies in order to demonstrate that, in osteoarthritis patients, both osteophytes formation and regenerative properties of muscle stem cells are related to the expression of BMP-2. In particular, we performed morphological and immunohistochemical studies to investigate muscle regeneration by satellite cells activity. Moreover, immunogold labeling allowed us to investigate the correlation between BMP-2 expression and the formation of stem cells syncytium and stem cells niche characteristics.

## 2. Material and Methods

In this study, we collected 40 muscle biopsies: 20 biopsies of osteoarthritic women (mean age 71.6 ± 10.3) who underwent a Total Hip Arthroplasty (THA) and 20 biopsies from osteoporotic women (mean age 82.4 ± 6.19) with a cervical femoral fragility fracture (Tables 1(a) and 1(b)). During open surgery for hip arthroplasty muscle biopsies were taken from the upper portion of the vastus lateralis muscle under patients' informed consent (approval reference number 85/12, independent ethical committee “Policlinico Tor Vergata”).

Exclusion criteria of the patients were history of neoplastic diseases, myopathies, or other neuromuscular diseases, assumption of antiosteoporotic drugs, or chronic administration of corticosteroid for autoimmune diseases (more than 1 month), diabetes, alcohol abuse, cigarettes smoking, and viral chronic infections (HBV, HCV, and HIV).

Patients were divided into osteoporotic (OP) and osteoarthrosic (OA) according to DEXA, *T*-score, and radiographic assessment by Kellgren-Lawrence scale. (Tables 1(a) and 1(b)).

In particular in OP group women with fragility hip fracture, a *T*-score ≤ −2.5 SD, and a negative radiographic framework for hip OA were included, whereas in OA group women with a positive radiogram for hip OA with a Kellgren-Lawrence score 3 or 4 and *T*-score ≥ −2.5 SD were included.

### 2.1. Bone Mineral Density Evaluation (DXA)

DXA was performed with a Lunar DXA apparatus (GE Healthcare, Madison, WI, USA). Lumbar spine (L1–L4) and femoral (neck and total) scans were performed, and BMD was analyzed as previously described [11]. Dual-energy X-ray absorptiometry measures BMD (grams/square centimeter) with a coefficient of variation of 0.7%. In the OA group, all measurements were performed on the nondominant side, while participants lay supinely on an examination table with their limbs abducted away from the trunk. For the OP group, BMD was measured on the limb opposite the fracture side. Results were expressed as absolute values. The Harris Hip Score (HHS) [12] of the affected side was calculated in all OA patients.

### 2.2. Histology

Muscle biopsies were fixed in 4% paraformaldehyde for 24 hours and paraffin embedded. Three-micrometer thick sections were stained with hematoxylin and eosin (H&E) and the pathological evaluation blindly was performed by two pathologists [13].

### 2.3. Morphometric Analysis

In order to assess fibers atrophy, a minimum of 200 muscle fibers per biopsy have been evaluated, comparing minimum transverse diameter and cross-sectional area of type I and type II fibers for relative prevalence. A threshold diameter lower than 30 *μ*m (minimum value of the normal range for women) characterized atrophic fibers [14, 15].

To calculate muscle areas H&E slides were scanned at 20x magnification by Iscan Coreo (Ventana, Tucson, AZ, USA). Areas of skeletal muscle and connective and adipose tissue were identified by a pathologist for each muscle biopsy and analyzed by Image Viewing software (Ventana, Tucson, AZ, USA).

### 2.4. Immunohistochemistry

Immunohistochemical characterization was performed to asses muscle fibers type, that is, fast and slow, the expression of myostatin, surface antigen CD44, and bone-muscle cross talk molecules such as BMP-2.

Briefly, 3-*μ*m-thick sections were pretreated with EDTA citrate pH 7.8 for 30 min at 95°C and then incubated, respectively, with mouse monoclonal anti-fast skeletal myosin for 60 min (1 : 100, clone MY-32, AbCam), mouse monoclonal anti-slow skeletal myosin for 60 min (1 : 100, clone NOQ7.5.4D, AbCam), rabbit monoclonal anti-myostatin for 45 min (1 : 100, clone ab134682, AbCam), rabbit monoclonal anti-CD44 for 60 min (predilute, clone SP37, Ventana, Roche). For BMP-2, 3-*μ*m-thick sections were pretreated with citrate pH 6.0 for 30 min at 95°C and then incubated with rabbit monoclonal anti-BMP-2 for 60 min (1 : 250 clone NBP1-19751, Novus Biologicals) [16]. Washing were performed with PBS/Tween 20 pH 7.6 (UCS diagnostic, Rome, Italy); reactions were revealed by HRP-DAB Detection Kit (UCS diagnostic).

### 2.5. Western Blotting

Biopsies from vastus lateralis muscle (pooled samples from 10 biopsies in each group) were homogenized in ice-cold lysis buffer. The homogenate was centrifuged at 12.000 ×g for 15 min at 4°C for isolation of total supernatant protein. Protein concentration was determined with Pierce BCA Protein Assay Kit (Thermo Scientific Pierce Protein Biology Products, Rockford, IL, USA) according to the instructions of the manufacturer. Forty *μ*g/lane was separated on 10 per cent SDS-PAGE and transferred to a polyvinylidene fluoride membrane (0.45 *μ*m, Immobilon-P transfer membrane, Millipore, USA). After blocking, the membranes were incubated overnight with a primary antibody for BMP-2 (1 : 500, clone NBP1-19751, Novus Biologicals), washed in TBST, and then incubated with HRP conjugated goat anti-rabbit IgG (UCS diagnostic) for 1 h. The immunoreactive signals were developed using an enhanced chemiluminescence kit (Millipore, USA) and exposed to Kodak film. The relative and normalized protein expression was calculated by GAPDH (glyceraldehyde-3-phosphate dehydrogenase). Intensity of western line was evaluated by ImageJ software.

### 2.6. Transmission Electron Microscopy (TEM)

Small samples of muscle tissue of each patient were fixed in 4% paraformaldehyde, postfixed in 2% osmium tetroxide, and dehydrated by a series of incubations in 30%, 50%, and 70% ethanol. Samples were then embedded in EPON resin (Agar Scientific, Stansted Essex CM24 8GF United Kingdom) [17], for morphological ultrastructural analysis, and in LR-White resin (Agar Scientific, Stansted Essex CM24 8GF United Kingdom), for ultrastructural immunohistochemistry. Tissues were cut [18] and stained with heavy metals solutions as described by Reynolds [19].

### 2.7. Immunogold Labeling

Ultrathin LR-White embedded sections, collected on formvar carbon-coated nickel grids, were incubated in drops of 1% bovine serum albumin (BSA) in phosphate-buffered saline (PBS) containing 0.02 M glycine and normal goat serum at room temperature for 30 min [20]. Sections were incubated overnight with a rabbit monoclonal anti-BMP-2 antibody (1 : 50 clone NBP1-19751, Novus Biologicals) at 4°C. After several washes with PBS + 0.1% BSA, grids were incubated with a 20 nm secondary antibody-gold particle complex (Agar Scientific, Stansted Essex CM24 8GF United Kingdom) at 1 : 10 diluted in PBS 0.1% BSA for 2 h at room temperature. After immunolabeling, sections were washed with PBS + 0.1% BSA, washed in distilled water, dried, and counterstained with uranyl acetate. All sections were examined by a Hitachi 7100 FA electron microscope.

### 2.8. Statistical Analysis

Statistical analysis was performed using GraphPad Prism 5 Software (La Jolla, CA, USA). Immunohistochemical data were analyzed by Mann-Whitney test (*p* < 0.0005).

## 3. Results

### 3.1. Fiber Composition and Incidence of Fiber Atrophy

**Figure 1:**
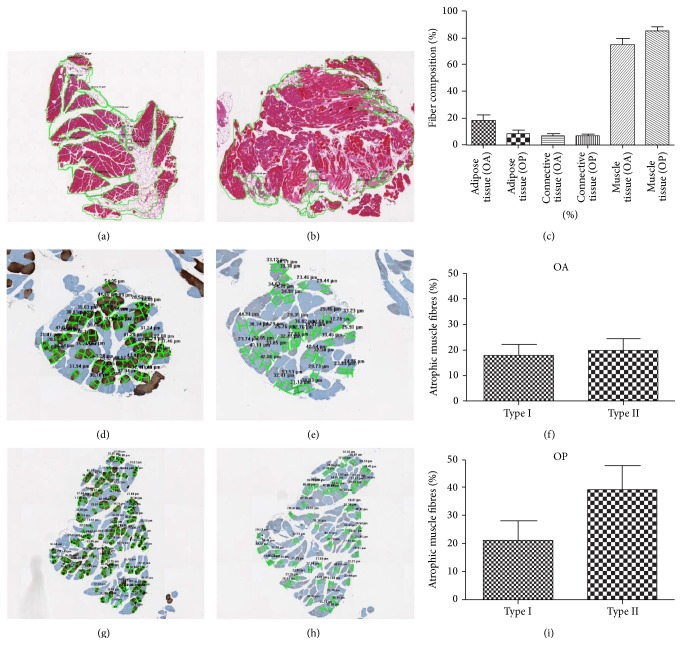
Muscle fibers composition and atrophy. ((a), (b), and (c)) In OA patients the rate of muscle loss was replaced mainly by adipose tissue (18.32%), whereas in OP muscle fasciculus atrophic muscle fibers were substituted by adipose (8.27%) and connective tissue (6.68%). ((d), (e), (f), (g), (h), and (i)) In OA patients we did not observed significant differences in fibers type atrophy, whereas In OP group we observed more than 50.00% of atrophic fibers with prevalence of type II fibers (21.10% type I and 39.20% type II).

Muscle fasciculus morphometric examination performed on digital scanned images showed a reduction of about 20% of muscle tissues in both OA and OP groups (Figures 1(a), 1(b), and 1(c)).

In OA patients muscle loss rate was replaced mainly by adipose tissue (18.32%) (Figure 1(a)), whereas in OP muscle atrophic fibers were substituted by a mixture of adipose (8.27%) and connective tissue (6.68%) (Figure 1(b)).

Slow myosin antibody and fast myosin antibody stains allowed us to discriminate type I and type II fibers, respectively. The morphometric analysis of muscle fibers in OA patients showed 38.00% of atrophic fibers with a diameter of less than 30 *μ*m (17.90% type I and 20.10% type II) (Figures 1(d), 1(e), and 1(f)). In OP group, we observed more than 50.00% of atrophic fibers with prevalence of type II fibers (21.10% type I and 39.20% type II) (Figures 1(g), 1(h), and 1(i)).

### 3.2. BMP-2 and Muscle Stem Cells Activity

Muscle biopsies of both OA and OP patients did not show peculiar signs of muscle pathology.

**Figure 2:**
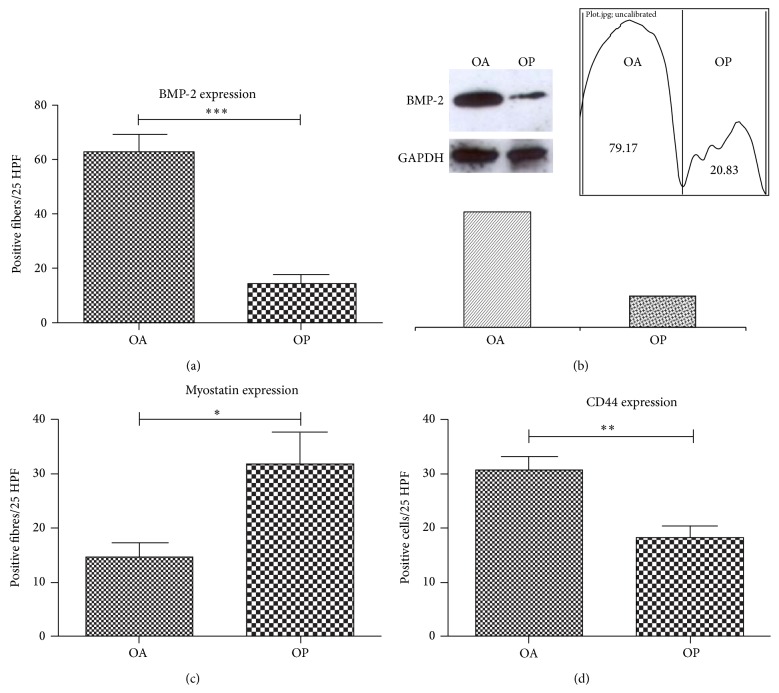
Protein expression analysis. Immunostaining for BMP-2, myostatin, and CD44 was evaluated by counting the number of positive fibers/cells on 25-high power field (HPF), whereas western blot for BMP-2 was evaluated by lines densitometry. (a) Notably, we found that OA muscle biopsies showed a significantly higher number of BMP-2-positive fibers (293.0 ± 35.4) as compared with muscle of OP patients (162.1 ± 33.7) (*p* < 0.0001). (b) Western blot lines show a higher expression of BMP-2 in OA patients compared to OP group. (c) The number of myostatin positive fibers in OP patients was significantly higher compared to OA group (*p* < 0.0200). (d) CD44 expression shows a significantly different rate of CD44 positive cells in OA group as compared with OP (*p* < 0.0020).

**Figure 3:**
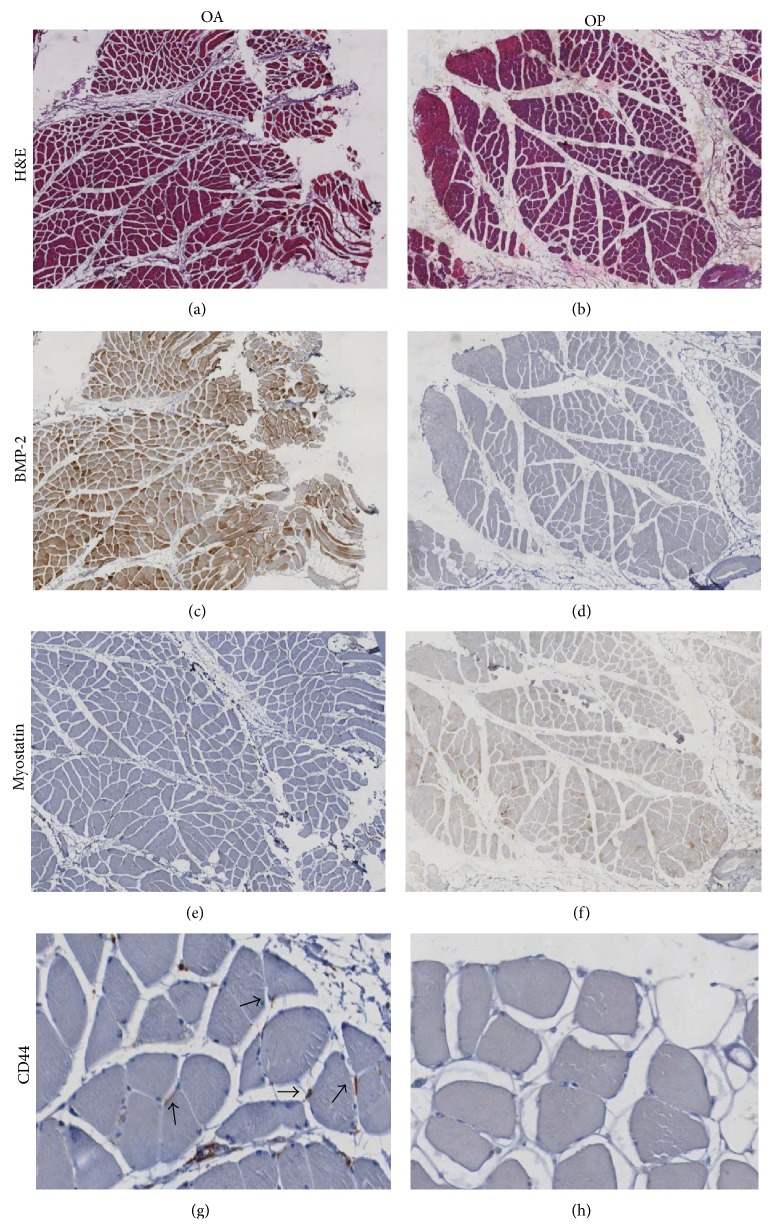
BMP-2 and satellite stem cells in muscle regenerations. ((a)-(b)) Hematoxylin and eosin sections of muscle biopsies showed a significant increase of fat tissue in OA (a) as compared to OP patients (b) (40x). (c) Image showed numerous BMP-2 positive fibers (40x). Often, in OP patients we did not observed BMP-2 expression (40x) (d). The Immunohistochemistry for myostatin was negative in OA muscle tissue (40x) (e). Muscle biopsies of OP group showed high/moderate expression of myostatin (40x) (f) inversely related to BMP-2 immunostain. Groups of satellite cells CD44 positive were focally dispersed in the tissue (200x) of OA patients (g) higher than that observed in OP patients (200x) (h).

BMP-2 expression was evaluated by counting the number of positive fibers on 25-high power field (HPF) (Figure 2(a)). BMP-2 positive fibers showed an intense staining in the perinuclear space and in the fiber body (Figure 3(c)). Notably, we found that OA muscle biopsies showed a significantly higher number of BMP-2-positive fibers (62.79 ± 6,205) as compared with muscle of OP patients (13.92 ± 3.343) (Figure 2(a)). Frequently, we observed BMP-2 positive fibers close to adipose tissue or to fiber degeneration areas (Figures 3(c) and 3(d)).

Western blot analysis of BMP-2, performed on 10 OA and 10 OP randomly selected samples, corroborated the immunohistochemical data. Indeed, we observed a higher BMP-2 expression in OA patients compared to OP groups (Figure 2(b)).

Muscle stem cells activity was studied by myostatin and CD44 expression. The number of myostatin positive fibers in OP patients was significantly higher compared to OA group (Figure 2(b)). Immunohistochemistry analysis showed that BMP-2 positive fibers were negative for myostatin and* vice versa* (Figures 3(e) and 3(f)).

Our results showed a significantly different rate of CD44 positive cells in OA group as compared with OP (Figure 2(c)). In particular, in OA patients many groups of CD44 positive satellite cells were focally observed in the tissue (Figures 3(g) and 3(h)).

### 3.3. Transmission Electron Microscopy (TEM)

**Figure 4:**
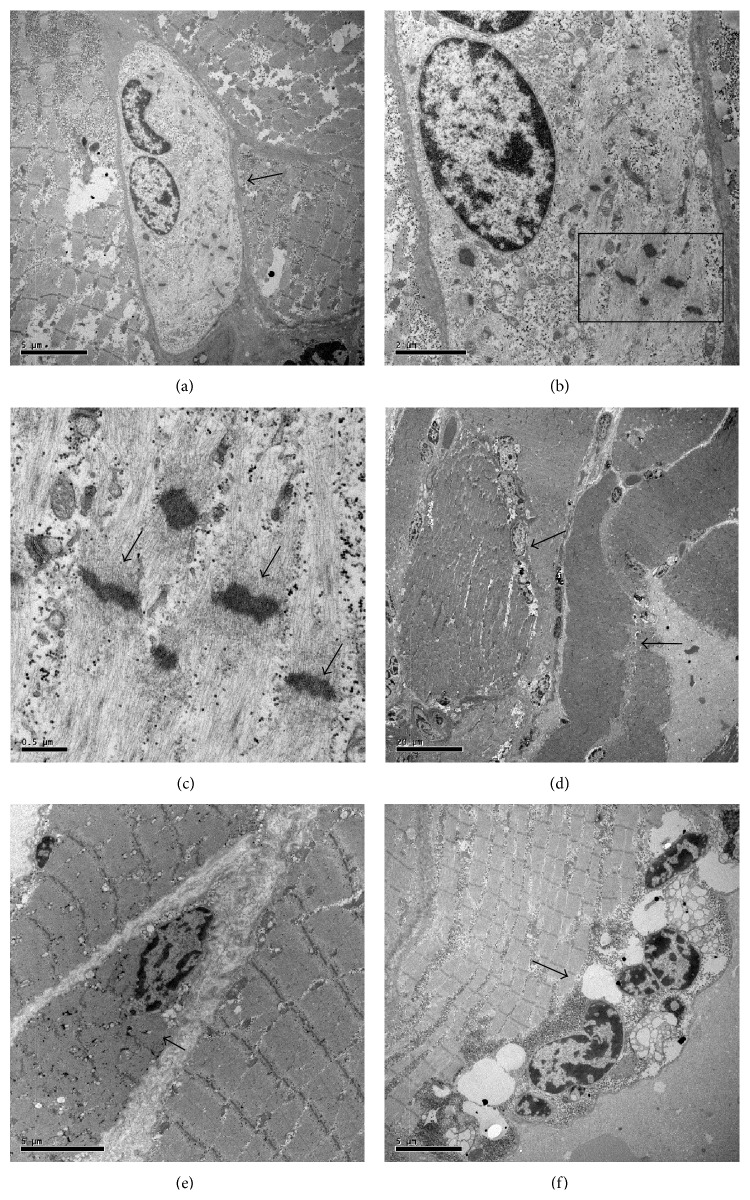
Transmission electron microscopy analysis of muscle stem cells. TEM analysis was performed to identify satellite cells according to their ultrastructural characteristics. In OA muscle biopsies we found several satellite cells generally placed between normal and degenerated fibers. In satellite cells syncytium (a) (arrow) we observed de novo production of sarcomeric structure (arrows) (10.000x and 50.000x) ((b)-(c)). TEM analysis of muscle biopsies from OP patients showed numerous degeneration areas (arrows) (2.500x and 5.000x) ((d)-(e)). Rare satellite cells syncytia (arrow) with obvious mark of degeneration, such as nuclear anomalies and vacuolations, we found in OP patients (5.000x) (f).

TEM analysis was performed to identify satellite cells according to their ultrastructural characteristics. In OA muscle biopsies we found several satellite cells generally located between normal and degenerated fibers (Figure 4(a)). Strikingly, these cells appeared to be tightly associated among them or fused to form a syncytium. Notably, in the cytoplasm of these cells we documented a* de novo* production of sarcomeric structures (Figures 4(b) and 4(c)). In OP patients we found numerous degeneration areas and immune cells infiltration (Figures 4(d) and 4(e)). The number of satellite cells was very low and their niches showed obvious mark of degeneration (Figure 4(f)).

### 3.4. Immunogold Labeling

In order to verify the correlation between BMP-2 and satellite cells activity we performed ultrastructural immunolabeling on both OA and OP muscle tissues.

**Figure 5:**
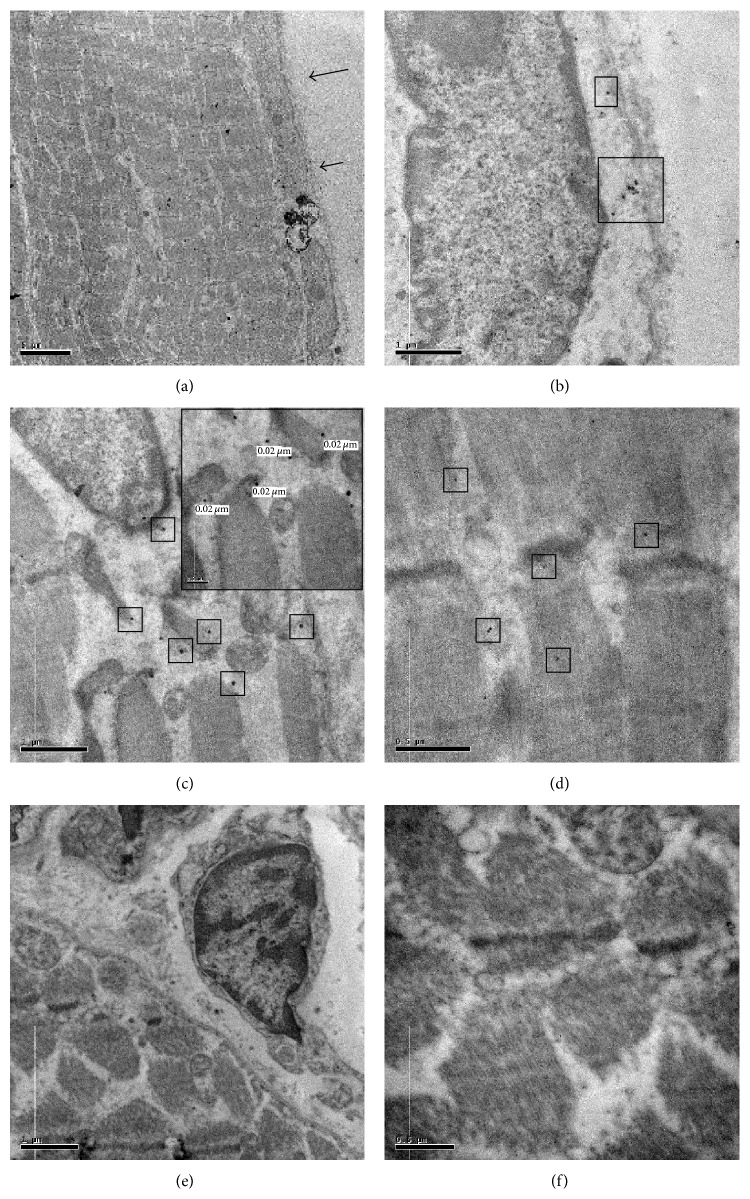
Molecular analysis of BMP-2 expression. In order to verify the correlation between BMP-2 and satellite cells activity we performed both western blot and immune-gold analysis. In muscle biopsies of OA patients we found several large satellite cells syncytium (arrows) (a). High magnifications show immunolabeling for BMP-2 in perinuclear areas (squares) (40.000x) (b). BMP-2 molecules were expressed in the satellite cells syncytium cytoplasm (squares) and next to mitochondria (insert) (40.000 and 60.000x) (c). Numerous BMP-2 molecules were found in the fiber body (40.000x) (d). OP patients do not express BMP-2 in satellite cells (10.000x and 40.000x) ((e)-(f)).

In OA group we found that satellite syncytium was more often associated with BMP-2 positive fibers whereas (Figure 5) we did not find any correlation between BMP-2 staining and satellite cells in muscles of OP patients. In particular, high magnification pictures show immunolabeling for BMP-2 in perinuclear areas (Figure 5(b)), next to mitochondria (Figure 5(c)) and in the fiber body (Figure 5(d)). The expression of BMP-2 demonstrated by an immunogold reaction at ultrastructural level in satellite cells syncytia of OA muscle biopsies suggest a strict correlation between BMP-2 expression and muscle regeneration capability.

## 4. Discussion

The hallmarks of osteoarthritis are degradation of cartilage matrix together with the production of new connective tissue in form of osteophytes on the joint surface or more notably at the joint margins. Inception* stimuli* responsible for osteophyte formation are not defined even though mechanical and humoral factors are probably involved. In experimental model of OA, injections of TGFb or BMP-2 in mice joints induced or enhanced osteophyte formation. Several studies investigated the role of BMPs as differentiation factors in bone tissue regeneration [21, 22]. Indeed, BMP-2 is immediately upregulated after bone fracture and it can be detected during the entire process of bone regeneration [23, 24]. Conversely, the role of BMPs in skeletal muscle physiopathology is still controversial. Ono et al. [25] sustained that myogenic precursor cells secreted BMPs (such as BMP-4) in order to inhibit the differentiation of satellite stem cells into new muscle fibers, whereas Sartori's group [26] established that BMP signaling is indispensable for positive regulation of adult muscle mass in normal and pathological situations.

In this study, we selected muscle biopsies from OA women who underwent a THA and OP women with a cervical femoral fragility fracture.

According to previous data [2], morphometric characterization of muscle biopsies demonstrated a higher percentage of atrophic fibers in OP patient with respect to OA group. As regards the tissue composition of muscle fascicles, OA patients were characterized by a higher fat substitution of muscle fibers. The presence of a larger amount of adipose tissue in OA subjects clinically corresponds to already mentioned increased BMI and to a higher functional impairment. Indeed, overweight and the consequent joint malalignment affect joints and accelerate their deformation and degeneration.

These preliminary clinical-morphometric data allowed us to hypothesize that BMP-2 expression in bone and skeletal muscle tissues of OA patients produce both osteophyte formation and muscle regeneration by satellite muscle cells.

Our results demonstrated that muscle tissues of aged OA patients frequently express BMP-2 close to degenerated areas or adipose tissue. Indeed, the expressions of these molecules were significantly higher in OA patients as compared to OP group.

Noteworthy, in our muscle biopsies, BMP-2 expression pattern was similar to that described in bone biopsies. Indeed, the bone microenvironment of OA patients is generally characterized by a 5-fold increase of BMP-2 [27]. Moreover, myofibrils with a strong positivity for BMP-2 inside the cell membrane do not express myostatin, a member of the TGF beta protein family that inhibits muscle differentiation and growth [28], and* vice versa*.

It is known that muscle satellite cells in adult act as a reserve population of cells able to proliferate in response to injury [29].

To better define a possible interplay between satellite cells activation and BMP-2 we investigated the CD44 receptor. This membrane molecule is involved in satellite cells migration, fusion, and syncytium forming capacity necessary for new muscle fibers formation [29]. Our results demonstrated a high number of satellite cells bearing CD44 antigen in OA patients. Altogether, these data, that is, high BMP-2, low myostatin, and high CD44 expression, suggest an active role of BMP-2 in muscle regeneration. To give strength to these immunohistochemical data we performed an ultrastructural study with immunophenotype characterization by immunogold labeling.

The ultrastructural analysis of tissues with a strong positivity for BMP 2 (OA patients) allowed us to confirm the presence of a high rate of both single satellite cells and satellite cells forming syncytium. Conversely, in muscle with low BMPs expression and muscle degeneration aspects (OP patients) we reported sparse, single, and not fused satellite cells. Moreover, the expression of BMP-2 demonstrated by ultrastructural immunogold reaction in the perinuclear area and in the fiber body of satellite cells syncytia suggests a strict correlation between BMP-2 expression and muscle regeneration capability.

Summing up, the higher BMP-2 expression in osteoarthritic patients could explain the increased bone mineral density as well as decreased muscle atrophy in osteoarthrosic patients. In particular, we demonstrate that BMP-2 induces skeletal muscle regeneration by activating satellite stem cells. This evidence lets us to hypothesize that endocrine failure of muscle tissue and bone mass diseases are strictly related.

## 5. Conclusions

The finding that both the activity of satellite muscle stem cells and bone remodelling are related to the same factors could shed new light on molecular mechanism of more relevant bone-muscle related diseases of the elderly, such as sarcopenia, osteoarthritis, and osteoporosis. From both diagnostic and therapeutic point of view, the control of physiological BMP-2 balance between bone and muscle tissue may be considered as a new pharmacological target.

## Figures and Tables

**(a):** 

Osteoporotic group				
Patient	Age	BMI	Menopause age	PASE test
1	88	19,06	47	45 (poor)
2	81	18,75	44	45 (poor)
3	93	25,48	46	50 (poor)
4	102	19,53	47	35 (inactivity)
5	84	27,34	40	95 (poor)
6	80	27,73	39	90 (poor)
7	86	26,67	43	40 (inactivity)
8	90	20,55	50	110 (moderate)
9	87	18,75	47	60 (poor)
10	86	17,78	46	40 (inactivity)
11	78	25,96	46	95 (poor)
12	80	24,14	33	90 (poor)
13	61	23,62	47	110 (moderate)
14	84	21,33	50	90 (poor)
15	79	28,71	52	100 (poor)
16	85	22,8	50	73 (poor)
17	81	24,12	42	90 (poor)
18	82	23,5	48	45 (poor)
19	85	22,8	45	85 (poor)
20	85	21,58	44	70 (poor)

**(b):** 

Osteoarthritic group					
Patient	Age	BMI	Menopause age	H.H. score	PASE test
1	76	30,44	52	42	106 (moderate)
2	83	20,03	50	40	100 (poor)
3	72	21,08	51	52	94 (poor)
4	51	34,72	51	32	130 (moderate)
5	79	28,04	47	26	90 (poor)
6	66	25,39	51	65	85 (poor)
7	78	32	52	55	50 (poor)
8	84	25,39	47	44	35 (inactivity)
9	78	22,04	55	47	110 (moderate)
10	69	24,34	50	29	65 (poor)
11	77	31,89	51	40	110 (moderate)
12	79	35,56	43	28	90 (poor)
13	83	23,63	50	85	130 (moderate)
14	84	27,73	45	26	90 (poor)
15	80	17,63	43	47	120 (moderate)
16	78	26,17	46	54	60 (poor)
17	68	24,61	52	66	110 (moderate)
18	64	22,1	41	55	90 (poor)
19	86	25,71	50	46	120 (moderate)
20	66	19,38	54	33	120 (moderate)
